# Poultry Slaughterhouse Wastewater Treatment Using Submerged Fibers in an Attached Growth Sequential Batch Reactor

**DOI:** 10.3390/ijerph15081734

**Published:** 2018-08-13

**Authors:** Hamidi Abdul Aziz, Nur Nasuha Ahmad Puat, Motasem Y. D. Alazaiza, Yung-Tse Hung

**Affiliations:** 1 School of Civil Engineering, Engineering Campus, Universiti Sains Malaysia, Nibong Tebal 14300, Penang, Malaysia; nurnasuhaahmadpuat@gmail.com (N.N.A.P.); my.azaiza@gmail.com (M.Y.D.A.); 2 Solid Waste Management Cluster, Science and Technology Research Centre, Engineering Campus, Universiti Sains Malaysia, Nibong Tebal 14300, Penang, Malaysia; 3 Department of Civil and Environmental Engineering, Cleveland State University, Cleveland, OH 44115, USA; yungtsehung@yahoo.com

**Keywords:** poultry slaughterhouse wastewater, sequential batch reactor, fiber, BOD, COD

## Abstract

In this study, a sequential batch reactor (SBR) with different types of fibers was employed for the treatment of poultry slaughterhouse wastewater. Three types of fibers, namely, juite fiber (JF), bio-fringe fiber (BF), and siliconised conjugated polyester fiber (SCPF), were used. Four SBR experiments were conducted, using the fibers in different reactors, while the fourth reactor used a combination of these fibers. The treatment efficiency of the different reactors with and without fibers on biochemical oxygen demand (BOD), chemical oxygen demand (COD), ammonia-nitrogen (NH_3_-N), phosphorus (P), nitrite (NO_2_), nitrate (NO_3_), total suspended solids (TSS), and oil-grease were evaluated. The removal efficiency for the reactors with fibers was higher than that of the reactor without fibers for all pollutants. The treated effluent had 40 mg/L BOD_5_ and 45 mg/L COD with an average removal efficiency of 96% and 93%, respectively, which meet the discharge limits stated in the Environmental Quality Act in Malaysia.

## 1. Introduction

Poultry slaughterhouses discharge a significant volume of highly polluted wastewater, principally during the slaughtering process and the periodic washing of residual particles, which cause a significant variation in the biodegradable organic matter concentration. Organic matter is considered the primary pollutant in the effluents of slaughterhouses [1]. The contribution of organic load to these effluents usually comes from different materials such as undigested food, blood, fat and lard, loose meat, paunch, colloidal particles, soluble proteins, and suspended materials [2,3]. Due to the mentioned components in the slaughterhouses wastewater, these wastewaters have a high concentration of organics such as chemical oxygen demand (COD), biochemical oxygen demand (BOD), phosphorous, and nitrogen [4]. Therefore, before discharging these wastewaters into receiving water bodies, an efficient treatment process should be carried out to prevent severe environmental pollution. In the last few decades, several treatment methods for the slaughterhouse wastewater have been reported. Biological (aerobic and anaerobic) treatment methods have been traditionally used for slaughterhouse wastewater treatment. However, both biological techniques have some limitations. For example, aerobic treatment processes require high energy consumption for aeration and generate a high amount of sludge [1]. The anaerobic treatment process of the poultry slaughterhouse wastewater is often impaired or slowed down because of the accumulation of suspended solids and floating fats in the reactor, which in turn leads to reduction in methanogenic activity and biomass washout [2]. Moreover, the anaerobic treatment process is more suitable in treating high organic loading wastewater [5,6].

Sequential Batch Reactors (SBR) are one of the biological processes applied to remove several types of pollutants. The SBR process is different from conventionally activated sludge techniques, because SBR merges all treatment units and operations into a single basin or tank, whereas traditional systems rely on various tanks. SBR has been successfully used for the treatment of domestic, municipal, industrial, dairy, synthetic, toxic and slaughterhouse wastewaters, swine manure, and landfill leachates [7,8,9,10,11,12].

Recently, the application of biomass carriers in the SBR process has been investigated by various researchers [13,14,15]. Fiber-based biomass carriers exhibit a good performance in removing pollutants, especially nitrogenous substances [16,17]. Previous studies that applied the swim bed technologies in SBR using bio-fringe (acryl fiber) revealed high treatment efficiency in removing pollutants, especially nitrogenous substances [17]. Several types of fibers have been used previously in wastewater treatments, such as plastic fibers [18,19], geotextiles [20], bio fringe acryl fiber [17], fibrous packing [21], and polyester fiber [22]. However, the application of fibers as attachment materials in SBR for poultry slaughterhouses wastewater treatment has not been well investigated.

The aim of this paper is to examine the potential use of various types of fibers as biomass carriers for slaughterhouses wastewater treatment by evaluating the removal efficiency of the pollutants with and without fiber in the reactor. The fibers involved are natural white Jute fiber (JF), synthetic siliconised conjugated polyester fiber (SCPF), bio-fringe (acrylic fiber) (BF), and the combination of three fibers in the reactor, called composite fiber (CF). The treatment efficiency of the different reactors with and without fibers on BOD, COD, ammonia-nitrogen (NH_3_-N), phosphorus (P), nitrite (NO_2_-N), nitrate (NO_3_), TSS, and oil-grease were evaluated. Parameters, such as BOD, COD, and NH_3_-N, were monitored every day during the experiments. However, the other parameters were evaluated based on the optimum value obtained.

## 2. Materials and Methods

### 2.1. Wastewater Source and Characteristics

The wastewater used in this study was collected from a local poultry slaughterhouse plant with a 13,000 birds per day capacity, located in the city of Nibong Tebal, Penang state, Malaysia, generating approximately 140 tons of wastewater daily. This wastewater, which is produced from different operations such as chickens cutting, chilling, scalding, packing and plant cleanup, was collected from the final collection tank after the screening of internal organs and feathers (partially treated using physical treatment). Wastewater samples of 150 to 200 L were collected twice per week, during the period from 23 May 2012 to 11 March 2013. Following the sampling procedure, the wastewater samples obtained were characterized based on pollutant concentration. Samples were preserved by storing in a cold room at 4 °C and were only taken out to room temperature 2 h before the experiment began. Characteristics of the raw wastewater are shown in Table 1.

### 2.2. Activated Sludge and Characteristics

The activated sludge (AS) used in this study was collected from the sludge dewatering system at the Jelutong Sewerage Treatment Plant (JSTP), Penang State, Malaysia. The AS in this study acts as microorganisms that are responsible for transforming the pollutants into acceptable end products. The AS also followed the poultry slaughterhouses wastewater storing procedures. Characteristics of the AS are shown in Table 1.

### 2.3. Fiber Preparation

Three types of fibers were used in this study as mentioned earlier. The first type was bio-fringe (BF) fiber made of acrylic fiber and imported from Japan. The other two types were Jute fiber (JF) and siliconised conjugated polyester fiber (SCPF). Composite fibers (CF) are a combination of these three fibers where all types were put together in the reactor. Both JF and SCPF were prepared similar to the size of the ready-made BF. The fibers were sewed neatly into pieces of yarns. Table 2 shows the physical properties of the fibers.

### 2.4. Reactor Setup

**Figure 1:**
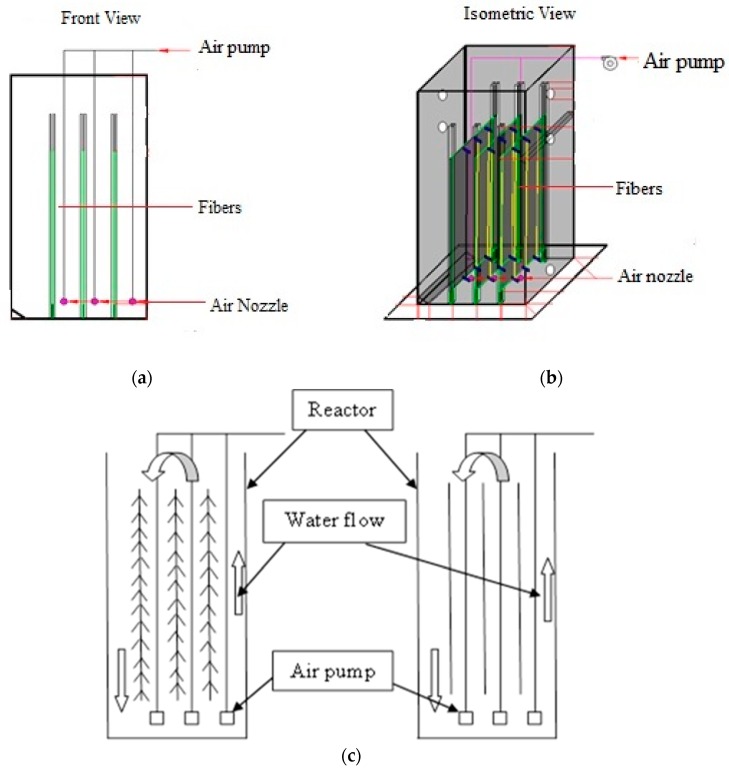
Schematic drawing of SBR reactor (**a**) Front view; (**b**) Isometric view; (**c**) Full details.

Two identical, laboratory scale Plexiglas reactors were used as SBR reactors for this study. Each reactor has the following dimensions: 80 cm × 40 cm × 25 cm with a total volume of 80 L. However, the experimental volume of the liquid for each reactor was 60 L. The first reactor was only operated with activated sludge without adding the fibers, while the other reactor was operated with activated sludge in the presence of fibers. Figure 1 shows the schematic diagram of the SBR reactor. The first cycle started with seeding of the AS collected from the JSTP. Following this, the reactor was fed with the collected raw poultry slaughterhouse wastewater during the filling phase and was aerated and mixed for a certain period of time during the aerating phase. The pH was adjusted approximately to 7.0 ± 0.5 and the mixed liquor suspended solids (MLSS) were maintained at a minimum range of 1500 mg/L to 4000 mg/L during the whole experiment. The adjustments were conducted before the aeration phase. The pH value was adjusted by adding either acid (0.5 M of H_2_SO_4_) or base solutions (0.5 M of NaOH). A 24-h cycle was selected, and the wastewater was operated for 20 h with the aeration rate of 60 L/min to make sure the wastewater and AS were mixed homogeneously. The final MLSS was 3782 mg/L. An air pump was used for the aeration and water circulation in the reactors. The aerated phase was stopped at the end of the aeration phase (after 20 h) and before the start of the settling phase (3 h). The decanting and discharging phase was the last process in the cycle, which meant that a cycle had been completed. After the first cycle was completed, the SBR reactor was filled with raw poultry slaughterhouse wastewater, aerated, settled, and decanted to repeat the second day treatment. Table 3 summarizes the operation design parameters of SBR reactor.

### 2.5. Operating Conditions

To maintain 1500 mg/L of MLSS, the poultry slaughterhouse wastewater feed was set at 21 L/day. The concentration of MLSS was checked during every aeration phase (1.00 p.m.). The ratio of food to microorganism (F/M) was set at 0.2, where F refers to BOD (mg) applied per day to the reactor and M refers to TSS (mg) in the reactor. The F/M ratio is an important parameter that represents the amount of substrate available for the microorganisms in activated sludge. A typical F/M ratio for SBR ranges from 0.04 to 0.1 [23], whereas for SBR nutrient removal it ranges from 0.2 to 0.6 [24]. Either a too low or too high value of F/M may cause filamentous bulking or foaming, which leads to poor settlebility. AS was added if the concentration of MLSS was below 1500 mg/L, which meant that the F/M ratio during the reactors operation was maintained below 0.25. Filamentous bulking might occur if the MLSS exceeded 4000 mg/L. In the case where the MLSS exceeded 4000 mg/L, some sludge would be wasted until the MLSS dropped to the desired level (1500–4000 mg/L). Meanwhile, the hydraulic retention time (HRT) and sludge retention time (SRT) have been calculated and kept at 72 h and 176 h, respectively. The reactors were operated at room temperature (25 °C) without any temperature controlling system. Both Filling and decanting processes were also conducted manually without any pumping system. Several experiment runs with the different types of fibers were carried out in order to achieve the objectives of the study. The filling period was 30 min, the aeration period was 20 h, and the settling period was 3 h.

### 2.6. Analytical Methods

In this study, the performance of the reactors was evaluated based on the values of BOD, COD, NH_3_-N, NO_2_, NO_3_, Phosphate, Oil-grease, TSS, and color. All of the mentioned parameters were determined and carried out as described in the Standard Methods for Water and Wastewater Examination [25]. The COD concentration was measured using a DR 2800 Spectrophotometer while NH_3_-N and PO_4_^3−^ concentration were calculated using a Hach DR 2500 Spectrophotometer. The removal efficiency of BOD, COD, and color was calculated using the following formula:

Removal Efficiency (%) = (*C_i_* − *C_f_*)/*C_i_* × 100 (1)

where *C_i_* and *C_f_* are the initial and final concentrations of parameters, respectively. Results from this study were analyzed using the Statistical Package for Social Sciences (SPSS) Version 17, via a one-way analysis of variance (One-Way ANOVA).

## 3. Results and Discussion

### 3.1. BOD and COD Removal

During the experiment, the pH of the activated sludge reactor was maintained at around 7 ± 0.5, the optimum range of pH for microbial growth. The MLSS was maintained in the system in the range between 1500 to 4000 mg/L.

The initial concentration of COD was 950 mg/L before treatment. Maximum removal efficiency of COD was achieved in day 12 with 69% and 293 mg/L COD. For the BOD value, the initial concentration was 350 mg/L where the maximum removal was achieved in day 13 with 88% removal efficiency with 105 mg/L BOD after treatment in the reactor without fibers. The growth of microbes started to become slow and stable (between days 11 to day 16) because the microbes did not have any shelter to regenerate before the cycle completes. As compared to the reactor with fibers, the reactor without fibers does not have shelter for microbes to attach.

On the other hand, after using the different types of fibers in the reactor, the removal efficiency of BOD and COD increased over the time in all reactors. As an overall result, the performance of SBR using fibers as an attachment material has demonstrated better results compared to SBR without fibers in the reactor. In general, the BOD removal for each type of fiber was efficient, with an average removal efficiency of higher than 90%. The observations showed that the CF reactor gives the higher removal efficiency of BOD with 96% (40 mg/L). For the JF and BF reactors, the optimum BOD removal was achieved on day 14 and day 12 with 62 mg/L (93%) and 45 mg/L (95%), respectively. Apart from that, the SCPF reactor showed optimum removal on day 13 with 50 mg/L BOD (94%).

**Figure 2:**
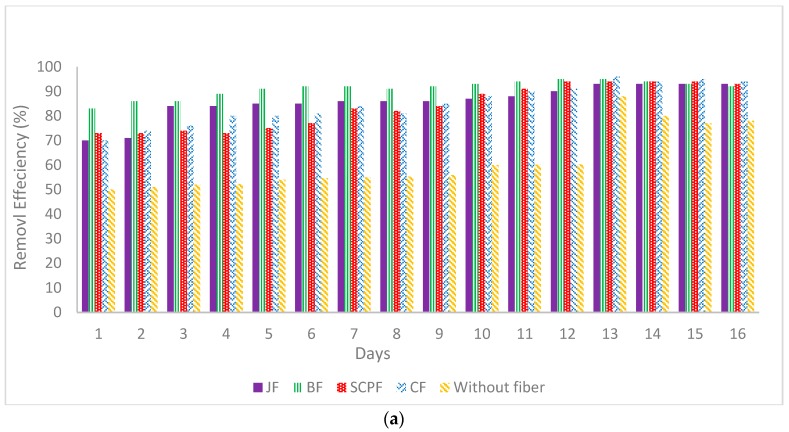

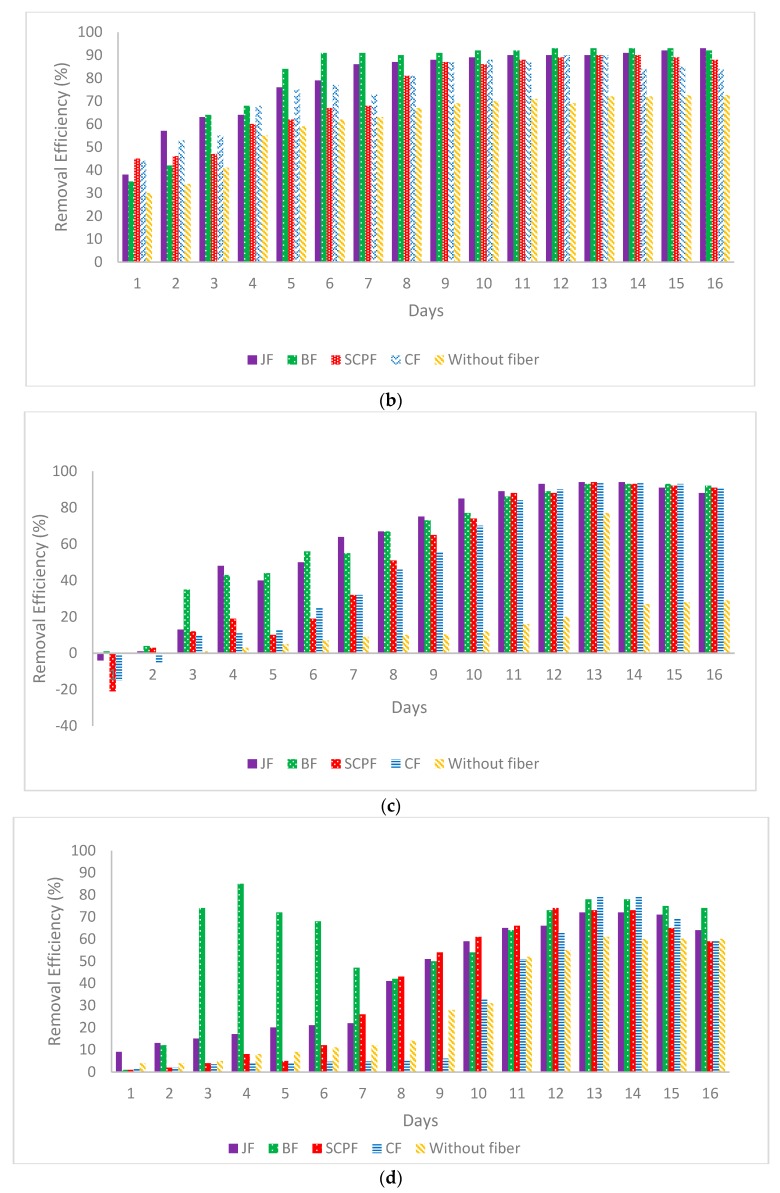

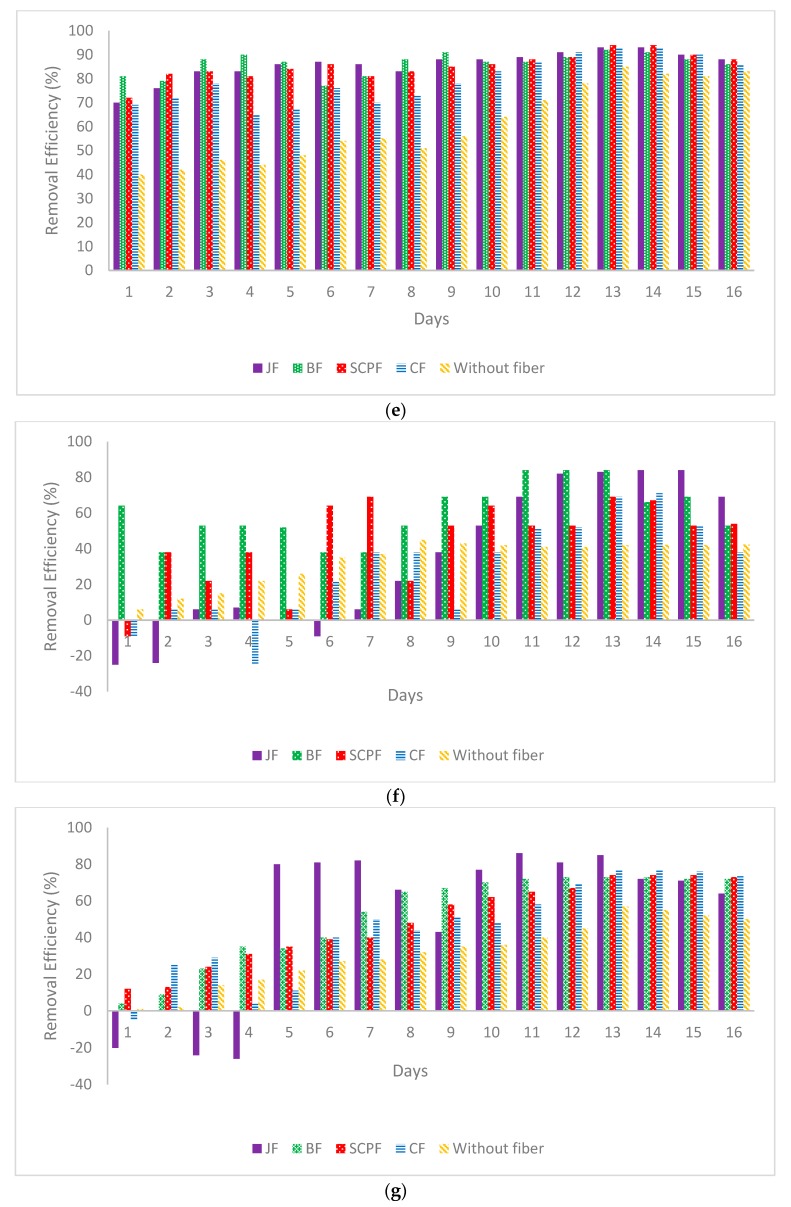

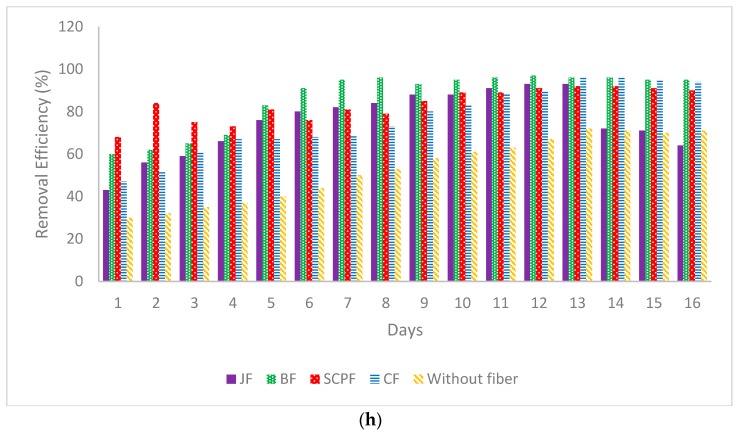
Daily monitoring for the removal efficiency of (**a**) BOD, (**b**) COD, (**c**) ammonia-nitrogen, (**d**) phosphate, (**e**) NO_2_, (**f**) NO_3_, (**g**) Oil-grease, (**h**) TSS, in all reactors.

For COD, the BF reactor showed a higher performance in the removal efficiency of COD with 93%. The COD removal achieved maximum removal on day 12 at 93% with 45 mg/L COD as shown in Figure 2.

### 3.2. Ammonia-Nitrogen Removal

The daily observations of the NH_3_-N showed that there was no removal for the first two days of treatment as shown in Figure 2. This observation was due to the increase in the concentration of ammonia-nitrogen due to the occurrence of nitrification [26]. According to the Fontenot et al. [27], in SBR, the nitrogen (protein or lipid) removal process was designed for the aerobic carbon removal and nitrification followed by an anoxic de-nitrification with the addition of an external carbon source.

Before using the fiber in reactors, the maximum removal was achieved on day 13, with the removal of 77% and concentration pollutant of 20 mg/L. It was observed that when the pH of the wastewater in the system was not basic, an oxidation of ammonia took place.

When the oxidation occurred, the pH of the wastewater quickly dropped, and this simultaneously produced nitrite. Ammonia can exist as molecular ammonia or ammonium gas. These two forms in water are strongly dependent on pH and temperature. Nitrification and de-nitrification occurred in good condition due to the aerobic and anaerobic zones inside the same system. The higher concentrations of ammonia were shown to have inhibitory effects of pH level during the anaerobic process [28].

On the other hand, and after using the fiber with SBR reactors, the NH_3_-N removal was considered to be high at 94%, 94%, 93% and 94% for CF, JF, BF and SCPF reactors, respectively. In the case of CF reactor, during the first and second day of treatment, no removal due to the increase in ammonia-nitrogen concentration from 85 mg/L to 98 mg/L (data not shown). However, in day 3 and 4 of treatment process, a fluctuation in removal efficiency was observed. The maximum removal efficiency was obtained at day 13 at 94%. The initial concentration of ammonia-nitrogen before treatment was 106 mg/L and diminished 5 mg/L after treatment. In general, biological nitrogen removal can be categorized into two separate steps: nitrification and denitrification. In the nitrification process, ammonium is usually converted to nitrate under aerobic condition, whereas the de-nitrification process converted the nitrate into nitrogen gas (N_2_) [29]. Therefore, when a higher aeration was used, a better removal efficiency of NH_3_-N was observed due to the fact that more DO was provided to the nitrifying bacteria in order to convert ammonium to nitrate.

### 3.3. Nitrite (NO_2_) and Nitrate (NO_3_) Removal

As can be seen in Figure 2, the maximum removal efficiency for NO_2_ was achieved on day 8 with the removal of 45% in the reactor without fiber. According to Erses et al. [28], when oxidation ammonia takes place, nitrite is produced simultaneously.

NH_4_^+^ + 1.5O_2_ → NO_2_^−^ + 2H^+^ + H_2_O (2)

However, the maximum removal efficiency for NO_3_ was achieved on day 13 with the removal of 85% when the remaining concentration in the reactor was 12 mg/L. After using the fibers in the reactors, the maximum removal efficiency for NO_2_ obtained in the JF reactor was 84%, which is approximately twice the removal efficiency of the reactor without fibers. In addition, the maximum removal efficiency for NO_3_ was 94%, which was achieved using SCPF reactor.

### 3.4. Phosphate Removal

The maximum removal efficiency for phosphate was achieved on day 13 with a removal efficiency of 61%, where the remaining concentration of 15 mg/L in the case of reactor without fiber, as shown in Figure 2. The removal showed a fluctuated trend on day 6, due to the increased concentration of nitrate in the anaerobic zone and phosphate in the aerobic zone (data not shown). The phosphorus content in the sample may also be affected by the apparatus used during the experiment if the apparatus was contaminated with detergent [30]. However, after using the fibers in reactors, the maximum removal efficiency of phosphate was 85% for the BF reactor during the experiment. No removal was obtained during the first day due to the increase of phosphate concentration from 39 mg/L to 65 mg/L. This was owing to the occurrence of the polyphosphate bacteria which started to accumulate large quantities of phosphate within their cells [31].

### 3.5. Oil-Grease and TSS Removal

In the reactor without fiber, the maximum removal efficiency for oil-grease was achieved on day 13 with 57% (117 mg/L). In addition, the same trend was observed for the TSS values. The maximum removal of TSS was recorded on day 13 with the remaining concentration at 72 mg/L (84%) from 532 mg/L. After applying the fibers in reactors, the maximum removal efficiency of the oil-grease and TSS was obtained using a JF reactor and BF reactor with 86% and 97% removal efficiency, respectively, as shown in Figure 2. The pattern of removal showed the fluctuated trend until day 4 of the treatment. Starting from day 5, the treatment slowly showed an increase in the removal over time. Maximum removal efficiency for oil-grease was obtained on day 11. The TSS removal efficiency showed an increase trend over that time. A maximum removal efficiency of TSS was achieved on day 13. The fluctuated trend that occurred in the early part of the treatment may be due to the fact that a pump was not used when the sample was taken. This led to the additional concentration of oil and grease and TSS. From the observation and the properties of the raw wastewater, the value of oil-grease increased because of not using the pump during the sample collection. The oil-grease may have been partially filtered using the pump.

### 3.6. Statistical Analysis

Table 4 summarized the maximum removal efficiency for all SBR. A One-way (ANOVA) test was used for multiple comparisons between the different types of the reactors. The test shows a significant difference between mean concentration BF and AS, CF, JF, and SCPF for all parameters tested. For example, for the BOD concentration after treatment, the ANOVA test shows that the concentration of BOD in the CF reactor was small as compared to the other reactors, which emphasize the experimental results. In addition, the BF reactor has a lower mean concentration for COD as compared to other reactors, which means that the BF reactor achieved the higher removal efficiency, proving the experimental results. For other parameters, similar results with experiments were obtained. The characteristics of final effluents for all reactors are summarized in Table 5.

## 4. Conclusions

This paper investigates the potential application of different types of fibers as biomass carriers in SBR reactors for slaughterhouses wastewater treatment, by evaluating the removal efficiency of the pollutants with and without fibers in a SBR reactor. The study showed that the removal efficiency of the SBR with fibers achieved a higher performance for all tested parameters as compared with the SBR without fibers. The SBR reactor with fibers as attachment materials enabled the attachment of suspended solids to the fibers, which increased the biomass concentration in the reactor and provided a better treatment efficiency. The treated effluent had 40 mg/L BOD and 45 mg/L COD with an average removal efficiency of 96% and 98%, respectively, which meet the discharge limits stated in the Environmental Quality Act in Malaysia. Moreover, all other parameters also satisfied the limits of the Environmental Quality Act in Malaysia.

## Figures and Tables

**Table 1:** Characteristics of raw wastewater and activated sludge (No. of samples = 7).

Parameter	Min	Max	Average	Std. Dev.
**Raw wastewater**				
BOD (mg/L)	573	1177	875	427.09
COD (mg/L)	777	1825	1301	741.04
NH_3_-N (mg/L)	56.7	104	80.35	33.44
Nitrite (mg/L)	45.3	80	62.65	24.53
Nitrate(mg/L)	52.6	178.4	115.5	88.95
Oil-grease (mg/L)	2361.5	3616	2988.75	887.06
TSS (mg/L)	395	783	589	274.35
pH	6.3	6.9	6.6	0.4242
**Activated sludge**				
BOD (mg/L)	1246	1548	1397	213.54
COD (mg/L)	51,248	59,345	55,296.5	5725.44
DO (mg/L)	0.65	0.68	0.665	0.0212
MLSS (mg/L)	47,000	59,000	53,000	8485.28
pH	6.75	6.85	6.8	0.0707

BOD: biochemical oxygen demand; COD: chemical oxygen demand; TSS: total suspended solids; DO: Dissolved oxygen; MLSS: Mixed liquor suspended solids.

**Table 2:** Fiber characteristics.

Characteristic	Types of Fibers			
	BF	JF	SCPF	CF
Support filament length (cm)	50	50	50	50
Yarn length (cm)	10 ± 0.5	10 ± 0.5	10 ± 0.5	10 ± 0.5
Yarn diameter (cm)	0.1–0.2	0.5–1.0	1.0–1.5	0.1–1.0
Yarn per string	65	20	20	20
Total weight (g)	19.3	52.65	42.15	114.1

BF: bio-fringe; JF: Jute fiber; SCPF: siliconised conjugated polyester fiber; CF: composite fibers.

**Table 3:** Summary of operating design parameters.

Parameters	Value
Volume (L)	80
Operating liquid volume (L)	60
Dimension (m)	0.4 × 0.4 × 0.25
Hours per cycle (h)	20
F/M ratio (day^−1^)	0.2
MLSS (mg/L)	1500
Feeding rate (L/day)	21
Hydraulic Retention Time (HRT) (days)	2.9
Sludge Retention Time (SRT) (days)	7.5
Temperature (°C)	25
Sludge Volume Index (SVI) (mg/L)	50

MLSS: mixed liquor suspended solids.

**Table 4:** Summary of maximum removal efficiencies for all reactors.

Max. Removal Efficiency (%)					
Parameters	Without Fiber	BF	JF	SCPF	CF
BOD	88	95	93	94	96
COD	69	98	93	91	90
NH_3_-N	77	93	94	94	96
Phosphate	61	85	72	74	79
NO_2_	45	81	84	69	71
NO_3_	86	92	93	94	93
TSS	84	97	93	92	96
Oil-grease	57	73	86	74	77

BF: bio-fringe; JF: Jute fiber; SCPF: siliconised conjugated polyester fiber; CF: composite fibers.

**Table 5:** Summary of final effluents of all parameters for all reactors.

Parameters	Without Fiber	BF	JF	SCPF	CF
BOD (mg/L)	120	70	65	65	65
COD (mg/L)	309	74	75	112	150
NH_3_-N (mg/L)	23	7	10	7	7.9
P (mg/L)	17	10	14	16	16
NO_2_ (mg/L)	51	30	20	37	40
NO_3_ (mg/L)	14	12	10	10	12
TSS (mg/L)	75	25	39	55	30
Oil-grease	855	801	431	751	723

BF: bio-fringe; JF: Jute fiber; SCPF: siliconised conjugated polyester fiber; CF: composite fibers.
